# Psychosocial and Body Image Variations in Professional Dancers: A Prospective Longitudinal Observational Study

**DOI:** 10.3390/sports14030099

**Published:** 2026-03-03

**Authors:** Marina Creazzo Maruschi, Gabriel de Souza Zanini, Pedro Luiz Santorsula de Paula Oliveira, Deivide Telles de Lima, Evandro Antônio Correa, Carlos Eduardo Lopes Verardi, Cátia Caldeira Ferreira, Víctor Hernández-Beltrán, José M. Gamonales, Mário Cunha Espada, Dalton Muller Pessoa Filho

**Affiliations:** 1 Department of Physical Education, Jahu Integrated Colleges (FIJ), Jau 17207-310, Brazil; marimaruschi@gmail.com (M.C.M.); gabriel.zanini@unesp.br (G.d.S.Z.); deivide-telles.lima@unesp.br (D.T.d.L.); prof.evandrocorrea@gmail.com (E.A.C.); 2 Post-Graduate Program in Movement Science, São Paulo State University (UNESP), Bauru 01049-010, Brazil; 3 Post-Graduate Program in Human Development and Technology, São Paulo State University (UNESP), Rio Claro 13506-900, Brazil; pedro.santorsula@unesp.br (P.L.S.d.P.O.); dalton.pessoa-filho@unesp.br (D.M.P.F.); 4 Post-Graduate Program in Education for Sciences, São Paulo State University (UNESP), Bauru 01049-010, Brazil; 5 Professional Post-Graduate Program in Physical Education in National Network (ProEF), São Carlos Federal University (UFSCAR), São Carlos 13565-905, Brazil; 6 Post-Graduate Program in Psychology in Development, São Paulo State University (UNESP), Bauru 01049-010, Brazil; carlos.verardi@unesp.br; 7 Physical Education Department, School of Sciences (FC), São Paulo State University (UNESP), Bauru 01049-010, Brazil; 8 Training Optimization and Sports Performance Research Group (GOERD), Faculty of Sport Science, University of Extremadura, 10005 Cáceres, Spain; catia.ferreira@ese.ips.pt (C.C.F.); martingamonales@unex.es (J.M.G.); 9 Escola Superior de Educação, Instituto Politécnico de Setúbal (CIEQV Setúbal), 2914-504 Setúbal, Portugal; mario.espada@ese.ips.pt; 10 Sport Physical Activity and Health Research & Innovation Center (SPRINT), Sport Sciences School of Rio Maior (ESDRM), Instituto Politécnico Santarém, 2040-413 Rio Maior, Portugal; 11 Faculty of Education and Psychology, University of Extremadura, 06006 Badajoz, Spain; 12 Instituto Universitario de Investigación e Innovación en el Deporte (INIDE), University of Extremadura, 10003 Cáceres, Spain; 13 Faculdade de Motricidade Humana, Centro Interdisciplinar de Performance Humana (CIPER), Universidade de Lisboa, 1499-002 Lisbon, Portugal; 14 Comprehensive Health Research Centre (CHRC), Universidade de Évora, 7000-645 Évora, Portugal

**Keywords:** professional dancers, psychophysiological balance, mood states, anxiety and stress, body image dissatisfaction

## Abstract

**Introduction:** Psychosocial functioning and body image are key dimensions of mental well-being and performance. Among professional dancers, competitive environments, aesthetic demands, and physical–emotional overload contribute to increased anxiety, stress, and mood disturbances, potentially impairing performance and heightening injury risk. **Objective:** To investigate longitudinal variations in psychosocial and emotional indicators among professional dancers throughout a season of rehearsals and performances. **Methods:** Thirteen dancers (9 women and 4 men) from a professional company were assessed across eight time points using the Brunel Mood Scale (BRUMS), State–Trait Anxiety Inventory (STAI-State), Recovery–Stress Questionnaire for Athletes (REST-Q 76 Sport), and Body Shape Questionnaire (BSQ). Data was analyzed using repeated-measures ANOVA with Bonferroni post hoc tests (*p* < 0.05). **Results:** Negative mood dimensions progressively increased (*p* < 0.01; η^2^p = 0.46, large), while vigor decreased (*p* = 0.03; η^2^p = 0.29, medium), indicating an inversion of the typical “iceberg” profile. Overall stress levels increased (*p* = 0.02; g = 0.53, power = 0.81) and perceived recovery declined (*p* = 0.04; g = 0.41, power = 0.78). State anxiety rose consistently (*p* < 0.01; η^2^p = 0.42), and body dissatisfaction, assessed via the BSQ, increased from “no concern” to “high concern” classifications (*p* = 0.03; g = 0.59, power = 0.84). **Conclusions:** Overall, the findings indicating a longitudinal pattern of increased psychometric strain indicators, inferred exclusively from psychometric trends, and conceptually consistent with a possible imbalance between perceived demands and perceived recovery, rather than reflecting objectively measured workload or recovery processes.

## 1. Introduction

Professional dance is characterized by a unique intersection of high physical demands, aesthetic rigor, and continuous psychosocial exposure, in which the body functions simultaneously as an instrument of artistic performance and an object of constant evaluation [1,2]. This dual role subjects’ dancers not only to intense physical training routines, comparable to those of high-performance athletes, but also to persistent identity-related pressures involving self-perception, body image, and social judgment [3,4,5]. According to previous literature, professional dancers are frequently exposed to prolonged rehearsal schedules, cumulative fatigue, and elevated emotional demands that extend beyond technical execution, particularly in environments characterized by perfectionism and strong aesthetic norms [6,7].

In broader conceptual terms, mental well-being has been defined by the World Health Organization (WHO) as a state in which individuals are able to recognize their abilities, cope with everyday stressors, and function productively within their social context [8]. Within the professional dance setting, however, mental well-being must be interpreted through the specific lens of artistic performance, where emotional regulation, interpersonal dynamics, and body-related perceptions are intrinsic components of occupational functioning [9,10]. Consequently, psychosocial functioning and body image emerge as central dimensions for understanding both performance quality and psychological vulnerability in this population.

Studies have shown that these pressures are associated with emotional overload, fatigue, pain, and exhaustion, often interpreted as intrinsic components of artistic commitment [6,7]. For example, Picard and Gaetz [11], in a qualitative study conducted with a specific sample of female ballet dancers, identified a cyclical trauma pattern marked by instability, lack of control, and perfectionism, alongside a high prevalence of body dysmorphia, eating disorders, anxiety, and depression. The competitive and evaluative nature of the dance environment may intensify psychological vulnerability, particularly under conditions of rigid aesthetic standards and constant external judgment, as reported in specific contexts and populations, potentially predisposing dancers to emotional exhaustion and mental health symptoms [1,2,12]. Additionally, Lopes et al. [13] reported a high prevalence of alexithymia and in a sample of professional contemporary dancers, suggesting that difficulties in emotional awareness may further exacerbate psychosocial distress within certain professional dance contexts.

Factors related to body image and satisfaction with one’s own appearance are widely recognized as relevant correlates of psychological functioning among dancers. Previous research has identified associations between body mass index and body dissatisfaction, which have been linked to the internalization of restrictive aesthetic ideals and heightened self-criticism within certain dance contexts [14,15]. More recent evidence suggests that the relationship between body image and eating-related pathology is complex and moderated by contextual and individual factors such as gender, dance discipline, and competitive level [16]. In highly aesthetic environments, greater body dissatisfaction has been empirically associated with lower self-esteem and poorer psychological well-being, even in the absence of clinically diagnosed eating disorders [14,15,16]. From a theoretical perspective, these mechanisms may also help explain potential negative implications for perceived performance and professional longevity, through increased self-criticism and emotional vulnerability. Furthermore, longitudinal evidence indicates that prolonged rehearsal periods and escalating performance demands are often accompanied by progressive increases in stress, particularly among female dancers, with implications for psychological balance and injury susceptibility [17]. From a psychophysiological perspective, sustained fatigue, reduced attentional capacity, and increased muscular tension under chronic stress may help explain how excessive workload contributes to greater injury vulnerability. Elevated stress levels have also been associated with a higher incidence of musculoskeletal injuries in dancers, especially affecting the lower limbs and lumbopelvic region [18,19,20,21].

Although previous studies have explored associations among mood states, anxiety, stress, recovery, and body image in dancers, most investigations remain limited to cross-sectional designs or isolated assessment points. Few studies have examined these variables in an integrated manner across an entire preparation and performance cycle using repeated measurements within the same professional cohort. This fragmentation in the literature limits the identification of critical periods of psychological vulnerability as well as potential windows for targeted intervention. Accordingly, the aim of this study was to longitudinally evaluate changes in mood, anxiety, stress, recovery, and body image among professional dancers across eight time points spanning the preparation, rehearsal, and performance phases.

## 2. Materials and Methods

This study employed a longitudinal observational design following the recommendations of Strengthening the Reporting of Observational Studies in Epidemiology (STROBE) reporting guidelines.

### 2.1. Setting and Participants

The study was conducted within a medium-sized professional dance company located in the interior of São Paulo, Brazil, whose repertoire primarily encompasses classical ballet and contemporary dance. The company employs approximately 20 professional dancers and also maintains educational activities for children and adolescents. The artistic season observed lasted nine months and included local performances as well as an international tour in Germany during the final phase of the cycle. This company was intentionally selected because it is the only professional dance company in the region that regularly participates in international artistic events and professional performances, representing the only feasible opportunity to conduct a longitudinal investigation involving an international performance cycle within our geographical and institutional context. It is important to emphasize that the season did not involve formal competitive ranking, adjudication panels, or external scoring systems. The term ‘high-demand context’ refers to professional exposure, selection processes, performance pressure, and artistic expectations rather than to formal competition.

Table 1 presents the demographic and anthropometric data of the study participants. A total of 13 individuals participated in the study (9 women and 4 men). Although the company employs a larger number of dancers, the present study specifically followed the subgroup of 13 dancers who were officially selected by the artistic direction to participate in the full preparation process and the international presentation in Germany. These individuals were those who effectively committed to the complete annual training and performance cycle associated with the international event, which justified their inclusion as the analytical cohort of this longitudinal study.

It is important to note that this selection process was based on artistic and organizational criteria defined by the company’s artistic direction (e.g., role allocation, technical and artistic suitability for the repertoire), and did not involve formal adjudication, ranking systems, or external competitive evaluation.

Rehearsals took place from Monday to Saturday, with intensity varying according to choreographic demands and proximity to performances. Participants engaged in one to two rehearsal and training sessions per day from Monday to Friday, in addition to one structured session on Saturdays, corresponding to approximately 10–18 h of structured training per week, with volume generally increasing as performances approached. Training intensity was not objectively monitored (e.g., no RPE, heart rate, session duration, or workload indices were collected) and was therefore not quantified in the present study. The characterization of workload is based on the reported training schedule rather than on physiological or perceptual load measures. Technical and repertoire classes were conducted in a wooden-floored studio of approximately 50 m^2^, equipped with fixed barres and wall-mounted mirrors. Ventilation and temperature control relied on fans and lateral windows, resulting in thermal fluctuations typical of the local climate. Psychometric assessments were administered in the main studio at standardized times before the start of rehearsals to minimize the influence of fatigue and acute training load.

The overall conditions of the season, including escalating technical demands, performance schedules, aesthetic expectations, and the emotional burden associated with the international tour, were described by the company and participants as progressively more demanding across the cycle. However, no objective indicators of training load or physiological stress were collected, and this contextual characterization is based solely on the reported structure of the season rather than on measured workload. This context justified the longitudinal design aimed at capturing temporal fluctuations throughout the entire artistic cycle. It is also important to note that most dancers did not rely exclusively on dance as their sole source of income and maintained parallel professional activities outside the company. This reality reflects the socioeconomic context of many professional dancers and represents an additional psychosocial demand relevant to the interpretation of the present findings.

All professional dancers active during the season were invited to participate through direct communication between the research team and the artistic direction during a scheduled meeting. Of the approximately 20 eligible dancers, 13 agreed to participate and met the inclusion criteria: (i) being an active professional dancer throughout the season, (ii) being at least 18 years old, and (iii) providing informed consent. No criteria related to minimum career duration, predominant dance style, or participation in specific performances were imposed. Exclusion criteria were: (i) absence in any of the assessment phases, (ii) incomplete psychometric data, and (iii) voluntary withdrawal during follow-up. No participant was excluded due to injury, and there were no refusals or dropouts across the eight assessment time points, resulting in a 100% retention rate among enrolled participants. However, as participation was voluntary, the possibility of selection bias cannot be excluded, and the psychological or physical characteristics of non-participants may have differed from those included in the study, which should be considered when interpreting the generalizability of the findings.

All 13 dancers completed the full set of psychometric evaluations across the eight predefined time points, with no sample loss or missing data. The complete follow-up of the same individuals across all assessments characterizes the study as a prospective longitudinal observational design with repeated measures. All participants signed an Informed Consent Form. The study protocol was approved by the Research Ethics Committee of the Integrated Colleges of Jahu (CAAE: 56989922.0.0000.5427), in accordance with the Declaration of Helsinki.

### 2.2. Instruments and Procedures

Assessments were conducted in loco at the company’s rehearsal studio. During an initial interview, sociodemographic and general health information were collected, followed by measurements of body mass and height using a calibrated anthropometric scale (Ramuza™, Santana do Parnaíba, SP, Brazil). Body mass index (BMI) was calculated according to the criteria established by the World Health Organization [22].

Psychometric data collection occurred at eight distinct time points throughout the season (pre-preparation, intermediate rehearsals, and performance periods). The instruments used were as follows: *Mood Profile (BRUMS—Brunel Mood Scale)* [23]—Adapted and validated for the Portuguese language by Rohlfs et al. [24]. The BRUMS evaluates six mood dimensions: tension, depression, anger, vigor, fatigue, and confusion. The sum of negative dimensions minus vigor yields the Total Mood Disturbance (TMD) score, an overall indicator of emotional balance. *State Anxiety Inventory (IDATE-E/STAI-STATE)*—Originally developed by Spielberger [25] and validated in Brazil by Biaggio and Natalício [26]. This instrument measures state anxiety, reflecting the intensity of apprehension, tension, and nervousness experienced at the moment of assessment. *Recovery-Stress Questionnaire for Athletes (REST-Q 76 Sport)*—Developed by Kellmann [27] and validated for the Brazilian sports context, this questionnaire assesses the balance between stress (general, specific, and global) and recovery (general, specific, and global), providing an index of the athlete’s psychophysiological recovery state. *Body Shape Questionnaire (BSQ)*—Proposed by Cooper et al. [28]. The BSQ evaluates body image perception and the degree of body dissatisfaction, considering self-deprecating thoughts and concerns about physical appearance. Higher scores indicate greater dissatisfaction with body image. For interpretative purposes, scores were classified according to established cut-off points: ≤80: no concern; 81–110: mild concern; 111–140: moderate concern; and ≥141: marked/high concern.

All instruments were administered by a single trained evaluator experienced in psychometric assessment, ensuring procedural standardization across all measurement points. Although this approach minimizes inter-rater variability, the possibility of evaluator-related bias cannot be entirely excluded, and no formal intra-rater reliability analysis was conducted across the eight assessment time points, which should be considered as a methodological limitation.

### 2.3. Statistical Analysis

All analyses were conducted using IBM Statistical Package for the Social Sciences (SPSS), version 26.0 (Chicago, IL, USA). Data distribution was examined using the Shapiro–Wilk test, selected for its sensitivity in small samples, and homogeneity of variances was assessed using Levene’s test. Given the repeated-measures structure of the dataset (eight time points per participant), two complementary analytical approaches were employed: repeated-measures ANOVA and Linear Mixed Models (LMM). Repeated-measures ANOVA was initially applied because the dataset contained complete observations for all participants across all assessment points, allowing traditional within-subject comparisons under balanced conditions. When significant main effects were observed, post hoc comparisons were adjusted using the False Discovery Rate (FDR) procedure. All *p*-values reported for pairwise comparisons in the Results section correspond to FDR-adjusted values.

Effect sizes for ANOVA were calculated using partial eta-squared (η^2^p) and interpreted according to Cohen’s criteria. In parallel, Linear Mixed Models were applied to provide a more robust evaluation of longitudinal trajectories. LMMs are particularly appropriate for repeated-measures observational data because they account for within-subject correlation and inter-individual variability without relying on the assumption of sphericity. In the present study, time was included as a fixed effect and participant ID as a random effect (random intercept). For LMM outcomes, standardized β coefficients, standard errors, Z-values, and *p*-values were reported. Temporal variation was additionally examined using linear trend components derived from LMM outputs.

Both analytical approaches yielded convergent conclusions across the main outcomes. Because LMMs offer greater flexibility and robustness for longitudinal data, interpretation of temporal patterns was primarily guided by the LMM estimates. Potential covariates (sex, age, and years of professional experience) were initially evaluated for inclusion in the Linear Mixed Models due to their known influence on psychological and behavioral variables in dancers. Each covariate was tested individually by adding it as a fixed effect to the base model (time as fixed effect and participant ID as random effect). Model fit was compared using changes in Akaike Information Criterion (AIC) and Bayesian Information Criterion (BIC), as well as by examining whether inclusion of the covariate meaningfully altered the fixed effect estimates for time. None of the tested covariates resulted in improved model fit or altered the interpretation of temporal trajectories; therefore, the final models were retained in their parsimonious form.

However, exploratory analyses indicated that none of these variables significantly improved model fit or altered temporal trajectories. Therefore, models were retained in their parsimonious form, including only time as a fixed effect and participant ID as a random effect, minimizing overfitting while preserving interpretability. No missing data were observed across the eight assessment points, as all 13 participants completed the full protocol. Consequently, no imputation procedures were required. As a sensitivity analysis, all models were re-run after excluding the highest and lowest baseline values for each outcome to verify that extreme scores were not driving the results. This procedure did not materially alter significance levels or effect size patterns.

Percentage changes between assessment points were calculated using the following formula: Δ% = [(baseline − follow-up)/baseline] × 100. The level of statistical significance was set at *p* ≤ 0.05.

## 3. Results

Significant differences between sexes were observed for body mass (men: 79.0 ± 7.12 kg; women: 58.3 ± 10.0 kg) and height (men: 1.84 ± 0.14 m; women: 1.64 ± 0.04 m); however, no significant difference was observed for BMI. The mean age of the participants was 30.7 ± 8.21 years, with no significant differences between groups (Table 1).

The repeated-measures ANOVA revealed a significant time effect for all negative mood dimensions: tension (F(7.84) = 2.87; *p* = 0.009; η^2^p = 0.19), depression (F(7.84) = 5.89; *p* < 0.001; η^2^p = 0.33), anger (F(7.84) = 5.75; *p* < 0.001; η^2^p = 0.32), fatigue (F(7.84) = 4.86; *p* = 0.012; η^2^p = 0.29), and confusion (F(7.84) = 4.34; *p* = 0.011; η^2^p = 0.27). The TMD also significantly increased (F(7.84) = 7.95; *p* < 0.001; η^2^p = 0.40), whereas vigor remained stable (*p* = 0.57) (Table 2). Post hoc tests (FDR correction) indicated that differences occurred mainly between the initial (1–2) and final (6–8) assessment points. All *p*-values reported for pairwise comparisons correspond to FDR-adjusted values. Significant increases were observed in tension (1–5, 1–6, 1–8, 2–8; *p* < 0.05), depression (1–4, 1–6, 2–7, 2–8; *p* < 0.01), and anger (1–8, 2–8; *p* < 0.001). Fatigue and confusion also showed significant differences between the beginning and end of the observation period (1–8; *p* < 0.05).

**Figure 1:**
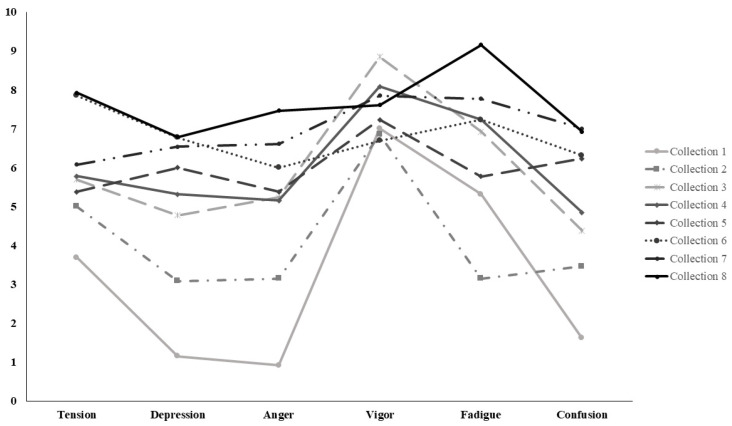
Iceberg Profile Obtained from the BRUMS Questionnaire Variables.

Linear mixed models showed significant positive linear trends across all negative mood dimensions, including tension (β = 0.49, 95% CI [0.24–0.74], *p* < 0.001), depression (β = 0.75, 95% CI [0.50–1.00], *p* < 0.001), anger (β = 0.59, 95% CI [0.52–1.04], *p* < 0.001), fatigue (β = 0.59, 95% CI [0.33–0.85], *p* < 0.001), and confusion (β = 0.74, 95% CI [0.47–1.01], *p* < 0.001). Vigor did not show a significant linear trend over time (β = 0.02, 95% CI [−0.21–0.26], *p* = 0.844). Total Mood Disturbance (TMD) also showed a significant positive linear trend (β = 3.33, 95% CI [2.44–4.22], *p* < 0.001) (Figure 1).

A significant time effect was observed for general stress (F(7.72) = 4.69; *p* < 0.001; η^2^p = 0.28), global stress (F(7.72) = 5.86; *p* < 0.001; η^2^p = 0.33), and specific stress (F(7.72) = 10.4; *p* < 0.001; η^2^p = 0.47). Post hoc analyses revealed marked increases between the initial and final phases: General stress: significant differences between collections 1–5, 1–7, 1–8, and 2–7 (*p* < 0.05); Global stress: significant differences between collections 1–7, 1–8, and 2–8 (*p* < 0.01); Specific stress: significant differences between collections 1–8, 2–4, and 3–8 (*p* < 0.01). Regarding recovery, a significant reduction was observed in general recovery (F(7.72) = 2.99; *p* = 0.012; η^2^p = 0.20) and specific recovery (F(7.72) = 2.51; *p* = 0.029; η^2^p = 0.17), whereas global recovery remained unchanged (*p* = 0.35). The LMM indicated a linear increase in specific stress (β = 0.23, 95% CI [0.14–0.31], *p* < 0.04), with no significant linear trends for the other dimensions (Table 3). Across the assessment period, higher mean values were observed in stress dimensions, alongside lower mean values in selected recovery dimensions.

**Figure 2:**
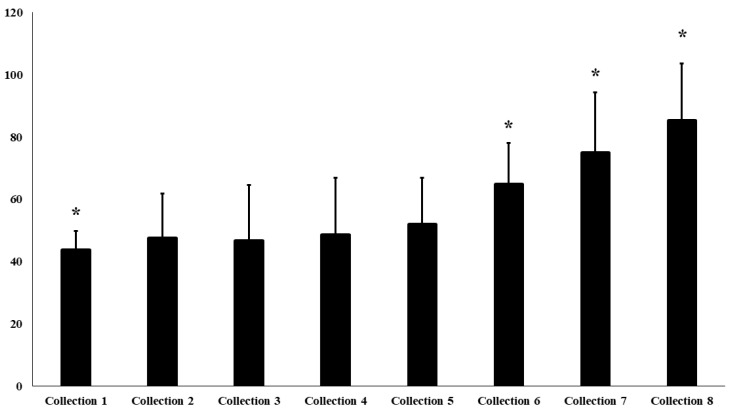
State Anxiety Level Assessed by the STAI-State Inventory. Legend: * denotes a statistically significant difference between collections (*p* < 0.05).

State anxiety levels showed a significant increase across the assessment points (F(6.72) = 2.66; *p* = 0.006; η^2^p = 0.05). Mean values rose from 43.7 ± 6.1 (“moderate” classification) at the first collection to 85.2 ± 18.5 (“high” classification) at the final one. Post hoc analysis revealed significant differences between collections 1–6, 1–7, and 1–8 (*p* < 0.01), with a positive linear trend (β = 1.54, SE = 0.944; Z = 2.014, 95% CI [0.04–3.05], *p* = 0.044) (Figure 2), state anxiety scores increased progressively across the assessment points.

**Figure 3:**
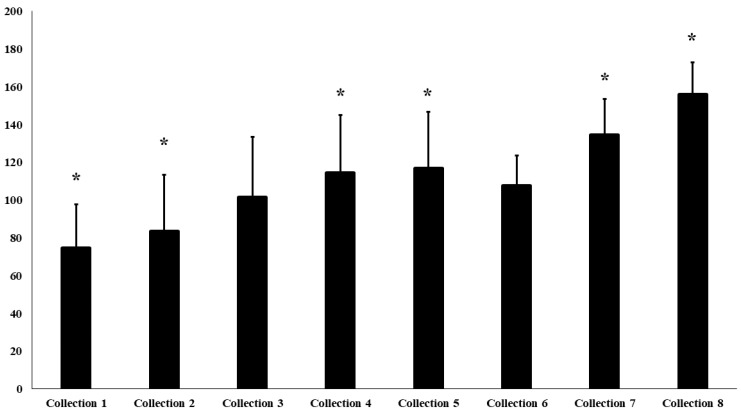
Level of Body Dissatisfaction Assessed by the BSQ. Legend: * denotes a statistically significant difference between collections (*p* < 0.001).

Mean scores on the Body Shape Questionnaire (BSQ) (Figure 3) increased significantly over the study period (F(6.72) = 4.03; *p* = 0.002; η^2^p = 0.23). The most pronounced differences occurred between the initial and final time points: collections 1–4, 1–5, 1–7, 1–8, and 2–4, 2–5, 2–7, 2–8 (*p* < 0.01). The mean score rose from 74.6 ± 23.1 (“no concern”) to 155.9 ± 17.2 (“high concern”), according to the standard BSQ classification thresholds (≤80 no concern; ≥141 high concern). The LMM confirmed an upward trend (β = 6.36, SE = 1.79, Z = 3.55, 95% CI [2.85–9.87], *p* < 0.001), showing progressively higher BSQ scores across the assessment points.

## 4. Discussion

The present study investigated longitudinal variations in behavioral variables among professional dancers throughout a complete preparation and performance cycle. The findings demonstrated a progressive increase in negative mood dimensions, heightened overall stress levels, continuous growth in state anxiety, and deterioration of body image. This set of results reveals a pattern of progressively unfavorable psychometric changes inferred from the observed psychometric trends, indicating a possible mismatch between increasing emotional and aesthetic demands and perceived recovery capacity across the observation period.

The mood alterations identified in this study are consistent with previous findings in sport psychology, which describe that intense training routines and proximity to competitive events are associated with increased tension, anger, and fatigue, along with reduced vigor [3,5,10]. Among dancers, these changes may be even more pronounced due to the overlap between physical load and performative demands in which the body simultaneously functions as both an instrument of performance and an object of aesthetic evaluation. In the present study, the company operated within a highly demanding and competitive artistic context, characterized by selective preparation processes and participation in international performances. In line with this context, previous research has shown that dancers in competitive environments tend to experience greater tension before performances and reduced pleasure afterward, suggesting that highly competitive artistic settings may attenuate some of the positive effects of dance on mood [29]. This pattern, also observed in athletes, has been associated with an increased risk of burnout, poorer artistic performance, and greater susceptibility to injuries [30,31,32].

These findings corroborate the results of the present study, in which the accumulation of stress and the reduction in perceived recovery over the weeks, as assessed through psychometric indicators, suggest a pattern that is conceptually consistent with a perceived load–recovery imbalance based on psychometric responses, in line with the psychobiological model proposed by Kellmann and Beckmann [33], although objective workload parameters were not directly quantified in the present study. This model suggests that when psychophysical load exceeds an individual’s adaptive capacity, symptoms such as fatigue, mood disturbances, and an increased risk of emotional exhaustion emerge. The systematic review by Glandorf et al. [34] reinforces this understanding, demonstrating that elevated stress levels are associated with higher rates of depressive symptoms, anxiety, and reduced vitality among athletes. Similarly, recent studies in dancers indicate that perceived autonomy support and healthy interpersonal relationships correlate with greater psychological satisfaction and positive affect, whereas ego-involving and conflictive environments promote a higher incidence of negative affect and emotional deterioration [30,35]. Taken together, these findings support the interpretation that sustained increases in stress indicators and reductions in perceived recovery may be associated with deterioration in emotional well-being, including fatigue, anxiety, and greater vulnerability to burnout. Conversely, autonomy support and positive interpersonal relationships appear to function as protective factors that promote psychological well-being. Overall, the available evidence highlights the importance of balancing demands and fostering healthy psychosocial environments to support both performance and mental health.

The progressive increase in anxiety observed among the dancers in this study aligns with the literature reporting persistently high levels of pre-performance and situational anxiety in this population [36,37]. This anxious response may be associated with continuous exposure to evaluative contexts and the internalization of self-image as a marker of competence, as proposed in the literature, potentially contributing to a psychophysiological cycle of stress and anticipatory concern. However, these contextual mechanisms were not directly assessed in the present study and should therefore be interpreted as theoretical explanations inferred from the observed psychometric patterns rather than empirically tested processes. Lopes et al. [13] reinforce this perspective by demonstrating a high prevalence of alexithymia and depression among contemporary dancers, suggesting that difficulties in recognizing and expressing emotions may be related to heightened anxious responses during periods of high-performance demand. Although such mechanisms were not measured here, the continuous increase in state anxiety observed across the assessment period may reflect both cumulative stress exposure and potential limitations in emotional regulation capacity, as discussed in previous studies.

Another relevant aspect concerns body dissatisfaction. Body Shape Questionnaire (BSQ) scores increased significantly across the observation period, indicating a worsening of body image concerns. This trend is consistent with the findings of Picard and Gaetz [11], who described a cyclical pattern of psychological vulnerability among female dancers characterized by perfectionism, body dissatisfaction, anxiety, and depressive symptoms. In the literature, such patterns have been discussed in relation to highly demanding training cultures and strong aesthetic norms commonly reported in classical dance contexts. Similarly, Boyes and Cornelissen [38] reported that dancers may internalize idealized body standards, which can be associated with greater body dissatisfaction and lower self-esteem. Drury et al. [37] also identified moderate-to-strong associations between body-checking behaviors and anxiety levels, supporting the conceptual link between self-image and emotional regulation processes. However, it is important to acknowledge that motivational climate, perceived aesthetic pressure, and related contextual mechanisms were not directly assessed in the present study. Therefore, these factors should be interpreted as plausible explanatory frameworks grounded in the previous literature rather than as empirically tested mechanisms within our data. Taken together, the findings suggest that increasing concerns about body image may be accompanied by broader psychological vulnerability in dancers. The available evidence highlights the importance of promoting educational and artistic environments that support healthy body image and emotional regulation, as a preventive strategy to protect psychological well-being in high-demand performance contexts [33,34,35,36].

The literature emphasizes that body dissatisfaction among dancers varies according to gender, style, and sociocultural context. Classic studies have already shown that adolescent ballerinas exhibit lower body satisfaction and a greater tendency toward negative body perception, with an increased risk of developing eating disorders [39], whereas more recent research demonstrates that professional ballerinas maintain high rates of body image distortion, affecting up to 40% of this group [40]. Consistently, Langdon and Petracca [41], and Swami and Tovée [42] observed that modern and street dancers show greater body appreciation and a lower drive for thinness, suggesting that contextual and cultural factors modulate body image perception.

The rise in TMD, anxiety, and body dissatisfaction scores observed in this study can be interpreted through the lens of the psychobiological model of stress vulnerability proposed by Markser [43]. From a theoretical perspective this model suggests that prolonged exposure to psychophysical demands in the absence of adequate recovery may be associated with neuroendocrine alterations, including possible hyperactivation of the hypothalamic–pituitary–adrenal (HPA) axis. It is important to emphasize, however, that no physiological markers (e.g., cortisol or other neuroendocrine indicators) were assessed in the present study, and therefore these mechanisms are discussed here as theoretical background rather than as empirical findings derived from our data. Within this conceptual framework, sustained psychophysiological strain has been linked in the literature to increased irritability, tension, and heightened self-critical processes, which may contribute to vulnerability to mood and body image disturbances. Recent studies also support this broader interpretation by showing that excessive body monitoring and frequent social comparison, features commonly described in professional dance environments, are associated with increased self-criticism and aesthetic dissatisfaction [44,45]. These mechanisms are presented strictly as theoretical interpretive frameworks and not as processes directly demonstrated by the present data.

Taken together, the results of this study support the notion that the increase in TMD, anxiety, and body dissatisfaction are not independent phenomena but interrelated expressions of continuous psychophysiological overload. This overload tends to perpetuate itself through a dysfunctional cycle in which perceived stress exacerbates negative effect, which in turn intensifies body image distortion and attentional focus on the body. Such a dynamic, described by Kellmann and Beckmann [33] and reinforced by Barrell and Terry [46], represents a possible pathway toward psychological overreaching and burnout, particularly in disciplines that combine high aesthetic demands, strict discipline, and constant public exposure.

Therefore, it becomes essential to implement preventive strategies for continuous psychometric monitoring and emotional support in high-performance artistic and athletic environments. Regular psychological support interventions, emotional regulation training, mindfulness sessions, and psychoeducational programs on body image can serve as protective mechanisms to mitigate chronic stress. Finally, it is recommended that future research expand sample size, integrate physiological indicators, and adopt experimental designs that allow investigation of the effects of intervention programs on the psychophysiological balance of dancers throughout the entire performance season.

Although the findings of the present study provide valuable insights into the psychosocial responses of professional dancers throughout a performance season, this study presents several methodological limitations that should be acknowledged. First, the small sample size (n = 13), inherent to the structure of professional dance companies, limits the statistical power and restricts the detection of subtle psychometric variations. Although all dancers completed every assessment point, the reduced sample constrains external validity and increases susceptibility to individual variability. Second, the absence of a control or comparison group prevents causal inference regarding the observed changes. Given that the psychological demands of dancers evolve simultaneously with artistic workload, rehearsals, and performance expectations, it is not possible to determine whether the patterns identified are specific to this company or reflect broader characteristics of the professional dance environment. Third, all measures were self-reported, which introduces the possibility of social desirability bias and emotional reactivity, particularly in contexts involving aesthetic judgement and performance pressure. Although validated instruments were used, the subjective nature of the constructs, mood, anxiety, stress–recovery, and body image, requires cautious interpretation. Fourth, the study did not assess contextual variables known to influence psychological well-being in dancers, such as perceived autonomy support, motivational climate, interpersonal relationships, and aesthetic pressure. These factors may mediate or moderate the associations observed and should be incorporated into future research. Finally, all psychometric instruments were administered by a single evaluator. While this ensured procedural standardization across all assessment points, it may also have introduced evaluator-related bias, which should be considered when interpreting the findings.

Finally, the results reflect the dynamics of a single professional company during one artistic season, including a specific repertoire, daily rehearsal load, and an international tour. These unique features may differ from the routines of other companies and limit generalizability. Despite these limitations, the study offers relevant practical insights for dance professionals, coaches, psychologists, and artistic directors. The progressive increase in stress, anxiety, and body dissatisfaction suggests the need for continuous psychometric monitoring throughout the season, rather than assessments restricted to performance periods. For future research, it should be considered that implementing structured mental-health programs (e.g., emotional-regulation training, mindfulness sessions, psychoeducation on body image, and regular feedback regarding recovery balance) may help mitigate perceived psychosocial strain. Companies may also benefit from integrating mental-health professionals into their artistic staff, promoting a supportive motivational climate, and creating spaces for dialogue about aesthetic pressure and emotional well-being. Such strategies can reduce vulnerability to burnout, preserve dancers’ psychological health, and potentially improve artistic performance and longevity in the profession.

## 5. Conclusions

The present study demonstrated progressive unfavorable longitudinal changes in psychometric indicators among professional dancers throughout the preparation and performance cycle, characterized by increased negative mood dimensions, elevated stress levels, heightened state anxiety, and worsening body dissatisfaction. These findings, inferred from longitudinal psychometric trends, suggest a pattern compatible with an imbalance between emotional, physical, and aesthetic demands and perceived recovery capacity, which may be associated with increased emotional strain and psychological vulnerability.

Taken together, the findings of the present study, interpreted in light of the existing literature, suggest the relevance of continuous psychometric monitoring and highlight the potential value of preventive strategies such as emotional regulation training, psychological support, and body image–focused approaches in high-demand performance contexts. However, it is important to note that no intervention was tested in the present study, and therefore these implications should be understood as theoretical and practical considerations rather than evidence of effectiveness. Overall, the findings contribute to advancing the literature on mental health and performance in high-demand artistic contexts and may offer useful perspectives for coaches, psychologists, and dance company directors when considering the development of support structures within professional environments.

Consequently, these findings open several avenues for future research aimed at improving mental health and performance in high-demand artistic contexts. These include investigating emotional regulation interventions, assessing continuous psychometric monitoring as a preventive tool, exploring the relationship between body dissatisfaction and artistic performance, and examining the role of resilience and social support as protective factors against chronic stress. Furthermore, it is relevant to compare different performing arts disciplines and to evaluate comprehensive prevention programs that combine psychological monitoring, body image education, and emotional regulation strategies. Such research may provide empirical evidence to support the development of healthier training and professional environments, ultimately optimizing both psychological well-being and artistic performance.

## Figures and Tables

**Table 1:** Sample Characterization at Baseline.

Variable	Sample (n = 13)	Male (n = 4)	Female (n = 9)	*p*
Age (years)	30.7 ± 8.21	28.0 ± 3.92	32.4 ± 10.7	0.925
Body mass (kg)	64.2 ± 6.32	79.0 ± 7.12	58.3 ± 10.0	0.001 *
Height (cm)	173 ± 5.11	184 ± 13.80	164.0 ± 3.89	0.001 *
BMI (kg/m^2^)	22.28 ± 3.27	23.16 ± 3.38	21.29 ± 3.34	0.556

Legend: kg: kilograms; cm: centimeters; BMI—Body Mass Index; * denotes a statistically significant difference between groups (*p* < 0.05).

**Table 2:** Repeated-Measures ANOVA Applied to the Mood Profile Variables Assessed by the BRUMS Test.

Variables	Tension	Depression	Anger	Vigor	Fatigue	Confusion	TMD
Collection 1	3.69 ± 1.21	1.15 ± 0.87	0.92 ± 0.12	7.00 ± 3.04	5.31 ± 1.97	1.62 ± 0.98	105.69 ± 8.01
Collection 2	5.00 ± 2.02	3.08 ± 1.22	3.15 ± 0.98	6.85 ± 2.97	3.15 ± 2.26	3.46 ± 3.93	111.01 ± 14.27
Collection 3	5.69 ± 1.45	4.77 ± 1.30	5.23 ± 1.49	8.85 ± 3.97	6.92 ± 1.66	4.38 ± 1.21	118.15 ± 18.26
Collection 4	5.78 ± 1.98	5.31 ± 1.65	5.15 ± 1.57	8.08 ± 2.81	7.23 ± 2.71	4.85 ± 2.31	120.15 ± 13.44
Collection 5	5.38 ± 1.65	6.00 ± 2.01	5.38 ± 2.31	7.23 ± 3.46	5.77 ± 2.11	6.23 ± 3.17	121.53 ± 14.51
Collection 6	7.85 ± 2.13	6.77 ± 2.34	6.00 ± 2.66	6.69 ± 2.15	7.23 ± 1.58	6.31 ± 2.63	127.46 ± 13.8
Collection 7	6.08 ± 1.87	6.54 ± 1.69	6.61 ± 1.97	7.85 ± 2.31	7.77 ± 1.37	7.00 ± 1.72	129.67 ± 12.65
Collection 8	7.92 ± 2.21	6.79 ± 3.26	7.46 ± 2.61	7.61 ± 2.75	9.15 ± 3.33	6.92 ± 2.90	130.61 ± 6.53
F	2.87	5.89	5.75	0.82	4.86	4.34	7.95
*p*-value	0.009 *	0.001 **	0.001 **	0.573	0.012 *	0.011 *	0.001 **
ƞ^2^p	0.193	0.324	0.329	0.06	0.288	0.265	0.398
β	0.49	0.75	0.59	0.02	0.59	0.74	3.33
SE	0.13	0.14	0.14	0.17	0.13	0.16	0.46
Z	3.75	5.45	5.75	0.14	4.42	4.62	7.28
*p*-value	0.001 **	0.001 **	0.001 **	0.887	0.001 **	0.001 **	0.001 **

Legend: TMD—Total Mood Disturbance (Ideal classification < 110). Collection 1—Baseline; Collection 8—Performance Session (Presentation in Germany); * denotes a statistically significant difference between collections (*p* < 0.05); ** denotes a highly significant difference between collections (*p* < 0.001). F = F statistic (ratio between explained variance and error variance); *p*-value = statistical significance level; η^2^p = partial eta squared (effect size, proportion of explained variance); β = regression coefficient (magnitude and direction of the effect); SE = standard error of the estimate; Z = standardized test statistic (coefficient divided by its standard error).

**Table 3:** Dimensions of Stress and Recovery Assessed by the REST-Q 76 Test.

Variables	General Stress	Specific Stress	Global Stress	General Recovery	Specific Recovery	Global Recovery
Collection 1	9.38 ± 3.48	11.00 ± 3.89	10.03 ± 3.89	9.46 ± 3.45	9.54 ± 3.26	9.77 ± 3.59
Collection 2	10.38 ± 2.24	10.15 ± 3.67	9.08 ± 3.77	10.64 ± 3.87	10.59 ± 3.78	10.36 ± 3.82
Collection 3	9.37 ± 3.11	11.21 ± 3.78	10.64 ± 2.89	9.49 ± 3.39	10.46 ± 3.45	10.24 ± 3.71
Collection 4	9.15 ± 3.67	11.34 ± 3.52	10.91 ± 3.56	9.23 ± 3.26	11.02 ± 3.89	9.62 ± 3.48
Collection 5	10.77 ± 3.59	10.97 ± 3.65	10.74 ± 3.87	9.69 ± 3.51	10.73 ± 3.26	9.38 ± 3.31
Collection 6	10.02 ± 3.89	10.65 ± 3.55	9.49 ± 3.48	9.46 ± 3.45	9.46 ± 3.41	10.31 ± 2.59
Collection 7	8.89 ± 3.48	9.31 ± 3.52	9.07 ± 3.48	8.54 ± 2.88	9.21 ± 2.87	10.00 ± 3.44
Collection 8	11.67 ± 3.56	12.99 ± 5.71	10.08 ± 4.98	7.11 ± 0.97	8.11 ± 1.46	11.66 ± 4.08
F	4.69	10.4	5.86	2.99	2.51	1.14
*p*-value	0.001 **	0.001 **	0.001 **	0.012 *	0.029 *	0.350
ƞ^2^p	0.036	0.027	0.030	0.014	0.027	0.004
β	0.044	0.228	0.085	0.148	0.044	0.033
SE	0.074	0.113	0.242	0.091	0.277	0.107
Z	0.60	2.02	0.35	1.63	0.16	0.31
*p*-value	0.55	0.043 *	0.73	0.101	0.087	0.761

Legend: Collection 1—Baseline; Collection 8—Performance Session (Presentation in Germany); * denotes a statistically significant difference between collections (*p* < 0.05); ** denotes a highly significant difference between collections (*p* < 0.001). F = F statistic (ratio between explained variance and error variance); *p*-value = statistical significance level; η^2^p = partial eta squared (effect size, proportion of explained variance); β = regression coefficient (magnitude and direction of the effect); SE = standard error of the estimate; Z = standardized test statistic (coefficient divided by its standard error).

## Data Availability

The data presented in this study are available on request from the corresponding author due to ethical restrictions related to data sensitivity.

## References

[1] Stutesman M., Goldstein T. (2023). Mechanisms for Affect Communication from Dance: A Mixed Methods Study. J. Creat. Behav..

[2] Lu Y. (2022). Analysis of Body and Emotion in Dance Performance. Proceedings of the 2021 Conference on Art and Design: Inheritance and Innovation (ADII 2021).

[3] Zanini G.D.S., Pessôa Filho D.M., Neiva C.M., Da Silva D.P., Ciolac E.G., Verardi C.E.L. (2018). Stress and Mood States Monitoring in a Swimming Team during a Competitive Period. J. Phys. Educ. Sport.

[4] Barreto P.M., De Moraes M.G., Zanini G.D.S., Neiva C.M., Terra G.D.S.V., Pessôa Filho D.M., Maffei W.S., Verardi C.E.L. (2016). Associated Factors between the State of Anxiety and a Specific Gymnastics Skill with Environmental Variations. J. Phys. Educ. Sport.

[5] Brandt R., Bevilacqua G.G., Crocetta T.B., Monteiro C.B.d.M., Guarnieri R., Hobold E., Flores L.J.F., Miarka B., Andrade A. (2021). Comparisons of Mood States Associated with Outcomes Achieved by Female and Male Athletes in High-Level Judo and Brazilian Jiu-Jitsu Championships: Psychological Factors Associated with the Probability of Success. J. Strength Cond. Res..

[6] Shaw J., Mattiussi A., Brown D., Springham M., Pedlar C., Tallent J. (2021). The Activity Demands and Physiological Responses Observed in Professional Ballet: A Systematic Review. J. Sport Exerc. Sci..

[7] Grove J., Main L., Sharp L. (2013). Stressors, Recovery Processes, and Manifestations of Training Distress in Dance. J. Danc. Med. Sci..

[8] Reed G.M. (2024). What’s in a Name? Mental Disorders, Mental Health Conditions and Psychosocial Disability. World Psychiatry.

[9] Song X. (2023). Research on the Influence of Psychological Quality on the Level of Athletes’ Competition and the Training Approach. Front. Sport Res..

[10] D’Alpino I.A., Moterosso J.P.C., Botaro W.R., Da Silva A.O.C., Sant’Anna P.G., Testa Junior A., Verardi C.E.L., Zanini G.S. (2022). Comparison between Mood States, Stress and Recovery in CrossFit^®^ Competitors and Non-Competitors. J. Phys. Educ. Sport.

[11] Picard M., Gaetz M. (2025). Cyclic Patterns of High-Risk Behaviours within Ballet Culture. BMC Psychol..

[12] Vasconcellos E.G., de Almeida A.R., Marimoto J.M. (2021). Comportamento Alimentar e Imagem Corporal de Bailarinos Profissionais Associados Às Percepções No Ambiente de Trabalho. RBNE-Rev. Bras. Nutr. Esportiva.

[13] Lopes C.P., Poussel M., Albuisson E., Temperelli M., Hily O., Chenuel B., Allado E. (2025). Prevalence of Alexithymia and Depression among Professional Contemporary French Dancers. Front. Sports Act. Living.

[14] Luna B.S.C., Ruíz E.J.C. (2021). Estima Corporal, Insatisfacción Corporal e Índice de Masa Corporal En Bailarinas de Ballet y Estudiantes. Psychol. Soc. Educ..

[15] Leal R.K.P., Souza N.S., Silva R.R.V. (2020). Autoestima e Satisfação Corporal de Bailarinas. RBONE-Rev. Bras. Obesidade Nutr. Emagrecimento.

[16] Li Q., Li H., Zhang G., Cao Y., Li Y. (2024). Athlete Body Image and Eating Disorders: A Systematic Review of Their Association and Influencing Factors. Nutrients.

[17] De Wet J.S., Africa E., Venter R. (2022). Recovery-Stress States of Professional Ballet Dancers during Different Phases of a Ballet Season. J. Danc. Med. Sci..

[18] Bickle C., Deighan M., Theis N. (2018). The Effect of Pointe Shoe Deterioration on Foot and Ankle Kinematics and Kinetics in Professional Ballet Dancers. Hum. Mov. Sci..

[19] Huang P.Y., Lin C.W., Jankaew A., Lin C.F. (2022). Relationship of Extrinsic Risk Factors to Lower Extremity Injury in Collegiate Ballet Dancers. Front. Bioeng. Biotechnol..

[20] Sun Y., Liu H. (2024). Prevalence and Risk Factors of Musculoskeletal Injuries in Modern and Contemporary Dancers: A Systematic Review and Meta-Analysis. Front. Public Health.

[21] Li F., Adrien N., He Y. (2022). Biomechanical Risks Associated with Foot and Ankle Injuries in Ballet Dancers: A Systematic Review. Int. J. Environ. Res. Public Health.

[22] Weir C., Jan A. (2019). BMI Classification Percentile and Cut Off Points.

[23] Terry P.C., Lane A.M., Fogarty G.J. (2003). Construct Validity of the Profile of Mood States—Adolescents for Use with Adults. Psychol. Sport Exerc..

[24] Rohlfs I.C.P.D.M., Rotta T.M., Luft C.D.B., Andrade A., Krebs R.J., De Carvalho T. (2008). Brunel Mood Scale (BRUMS): An Instrument for Early Detection of Overtraining Syndrome. Rev. Bras. Med. Esporte.

[25] https://journal.sipsych.org/index.php/IJP/article/view/620.

[26] Biaggio A.M.B., Natalício L. (1979). Manual Para o Inventário de Ansiedade Traço-Estado (IDATE).

[27] Kellmann M., Kallus K.W., Samulski D., Costa L., Simola R. (2009). Questionario de Estress e Recuperacao Para Atletas [The Recovery-Stress Questionnaire for Athletes].

[28] Cooper P.J., Taylor M.J., Cooper Z., Fairbum C.G. (1987). The Development and Validation of the Body Shape Questionnaire. Int. J. Eat. Disord..

[29] Zajenkowski M., Jankowski K.S., Kołata D. (2015). Let’s Dance—Feel Better! Mood Changes Following Dancing in Different Situations. Eur. J. Sport Sci..

[30] Almásy C., Fedor A.R. (2025). Psychosocial Aspects of Injuries Among Professional Folk Dancers. Int. J. Environ. Res. Public Health.

[31] Dwarika M.S., Haraldsen H.M. (2023). Mental Health in Dance: A Scoping Review. Front. Psychol..

[32] Yu H., Teo E.W., Tan C.C., Chang J., Liu S. (2025). Exploring the Factors Associated with Professional and Non-Professional Dancer Well-Being: A Comprehensive Systematic Review. Front. Psychol..

[33] Kellmann M., Beckmann J. (2018). Sport, Recovery, and Performance.

[34] Glandorf H.L., Madigan D.J., Kavanagh O., Mallinson-Howard S.H. (2025). Mental and Physical Health Outcomes of Burnout in Athletes: A Systematic Review and Meta-Analysis. Int. Rev. Sport Exerc. Psychol..

[35] Quested E., Duda J.L. (2010). Exploring the Social-Environmental Determinants of Well- and Ill-Being in Dancers: A Test of Basic Needs Theory. J. Sport Exerc. Psychol..

[36] Berndt C., Strahler J., Kirschbaum C., Rohleder N. (2012). Lower Stress System Activity and Higher Peripheral Inflammation in Competitive Ballroom Dancers. Biol. Psychol..

[37] Drury C.R., Armeli S., Loeb K.L. (2024). Body Checking and Avoidance among Dancers. Eat. Behav..

[38] Boyes J.E., Cornelissen K.K. (2024). The ‘Ideal’ Dancer: An Investigation into Predictors of Body Image Dissatisfaction among Male Dancers, Female Dancers and Their Non-Dancing Counterparts. PLoS ONE.

[39] Bettle N., Bettle O., Neumärker U., Neumärker K.J. (2001). Body Image and Self-Esteem in Adolescent Ballet Dancers. Percept. Mot. Ski..

[40] Da Silva C.L., De Oliveira E.P., De Sousa M.V., Pimentel G.D. (2015). Body Dissatisfaction and the Wish for Different Silhouette Is Associated with Higher Adiposity and Fat Intake in Female Ballet Dancers than Male. J. Sports Med. Phys. Fit..

[41] Langdon S.W., Petracca G. (2010). Tiny Dancer: Body Image and Dancer Identity in Female Modern Dancers. Body Image.

[42] Swami V., Tovée M.J. (2009). A Comparison of Actual-Ideal Weight Discrepancy, Body Appreciation, and Media Influence between Street-Dancers and Non-Dancers. Body Image.

[43] Markser V.Z. (2011). Sport Psychiatry and Psychotherapy. Mental Strains and Disorders in Professional Sports. Challenge and Answer to Societal Changes. Eur. Arch. Psychiatry Clin. Neurosci..

[44] Kalyva S., Yannakoulia M., Koutsouba M., Venetsanou F. (2023). Disturbed Eating Attitudes, Social Physique Anxiety, and Perceived Pressure for Thin Body in Professional Dancers. Res. Danc. Educ..

[45] Ohashi Y.B., Wang S.B., Shingleton R.M., Nock M.K. (2023). Body Dissatisfaction, Ideals, and Identity in the Development of Disordered Eating among Adolescent Ballet Dancers. Int. J. Eat. Disord..

[46] Barrell G.M., Terry P.C. (2003). Trait Anxiety and Coping Strategies among Ballet Dancers. Med. Probl. Perform. Artist..

